# The Arabidopsis-Trichoderma interaction reveals that the fungal growth medium is an important factor in plant growth induction

**DOI:** 10.1038/s41598-018-34500-w

**Published:** 2018-11-06

**Authors:** Enrique González-Pérez, María Azucena Ortega-Amaro, Fatima Berenice Salazar-Badillo, Elihú Bautista, David Douterlungne, Juan Francisco Jiménez-Bremont

**Affiliations:** 1 Laboratorio de Biotecnología Molecular de Plantas, División de Biología Molecular, San Luis Potosí, Mexico; 2 CONACYT-Consorcio de Investigación, Inovación y Desarrollo para las Zonas Aridas (CIIDZA), Instituto Potosino de Investigación Científica y Tecnológica A. C., San Luis Potosí, S.L.P. Mexico; 3 Catedrático CONACYT – Instituto Potosino de Investigación Científica y Tecnológica A.C. (IPICyT), División de Ciencias Ambientales - Camino a la Presa San José 2055. Col. Lomas 4 sección CP. 78216., San Luis Potosí, S.L.P. Mexico

## Abstract

Trichoderma spp colonizes the plant rhizosphere and provides pathogen resistance, abiotic stress tolerance, and enhance growth and development. We evaluated the Arabidopsis-Trichoderma interaction using a split system in which *Trichoderma atroviride* and *Trichoderma virens* were grown on PDA or MS medium. Arabidopsis growth was significantly increased at 3 and 5 days post-inoculation with both Trichoderma species, when the fungal strains were grown on PDA in split interaction. The analysis of *DR5:uidA* reporter line revealed a greater auxin accumulation in root tips when the fungi were grown on PDA in a split interaction. The root hair-defective phenotype of Arabidopsis *rhd6* mutant was reverted with both Trichoderma species, even in split interactions. At 12 °C, Trichoderma species in split interactions were able to mitigate the effects of cold stress on the plant, and also Trichoderma induced the *AtERD14* expression, a cold related gene. Volatile organic compounds analysis revealed that Trichoderma strains produce mainly sesquiterpenes, and that the type and abundance of these compounds was dependent on the fungal strain and the culture medium. Our results show that fungal nutrition is an important factor in plant growth in a split interaction.

## Introduction

Plant development is influenced by environmental factors such as water, light, temperature, nutrients, and microorganisms. Several studies have reported on the beneficial effects of some microorganisms on plants^1^. It has also been reported that plant roots secrete several compounds such as sugars, organic acids, amino acids, and phenolic compounds that stimulate the beneficial relationship between plants and microbes^2^. The basis of the molecular mechanisms underlying the relationship between plants and microbes could be found in the genomes of both organisms. This symbiosis can be orchestrated by complex changes in the gene expression that give rise to modulation of biochemical, hormonal, and metabolic pathways in both the plant and microorganism^3,4^.

*Trichoderma spp* are soil-borne fungi that have developed the ability to establish a beneficial symbiosis with plants. It has been reported that during the plant-Trichoderma interaction, the fungus can benefit the plant in different ways, for example by increasing biomass, nutrients intake, tolerance to different types of biotic and abiotic stresses, and even enhancing the germination rate^5–9^.

Trichoderma species are prolific producers of many small secondary metabolites, some of them with an agricultural application, such as indole-3-acetic acid (IAA), indole-3-acetaldehyde (IAAld), indole-3-ethanol (IEt) and siderophores^10,11^. Likewise, it has been shown that the production of volatile organic compounds (VOCs) by Trichoderma and some beneficial rhizobacteria are related to an increase in plant growth^12–16^. Recently, a Trichoderma-plant interaction in the absence of physical contact between the organisms looks highly attractive due to the beneficial effects of volatile organic compounds (VOCs), which are produced by the microorganism during the interaction with the plants. It has been reported that these VOCs have the ability to induce plant growth, and also to stimulate the plant defense towards attacks by pathogenic microorganisms, and more recently to increase tolerance towards salt stress^8,15,17,18^.

Previously we reported that *T. atroviride* and *T. virens* enhance plant growth^9^, in particular we stated that this plant growth effect is potentiated when the interaction occurred without physical contact. Nevertheless, in this split interaction Trichoderma species were grown on Murashige-Skoog (MS) medium, a conventional culture medium for Arabidopsis^9^. Here, we analyzed the Arabidopsis growth promotion with Trichoderma grown in potato dextrose agar (PDA), an optimal fungal culture medium. We used the split growth system to examine the interaction between Arabidopsis plantlets and *T. virens* or *T. atroviride* at 12 and 22 °C. In the split Arabidopsis-Trichoderma interaction, the growth promotion induction of Trichoderma strains growing in MS and PDA media was compared. We found that Trichoderma grown on PDA showed the greatest positive impact on Arabidopsis seedlings growth. The auxin responses were assessed using the *DR5::uidA* reporter line and *rhd6* mutant in direct and split interactions. Besides, we analyzed the volatile organic compounds (VOCs) profiles emitted by *T. atroviride* and *T. virens* when the fungi were grown in split interaction on MS or PDA media. Based on the data reported below, we show that fungal nutrition is an important factor in promoting the growth of Arabidopsis in a split interaction.

## Results

### The improvement of Arabidopsis growth in a Trichoderma split interaction is influenced by PDA medium

**Figure 1:**
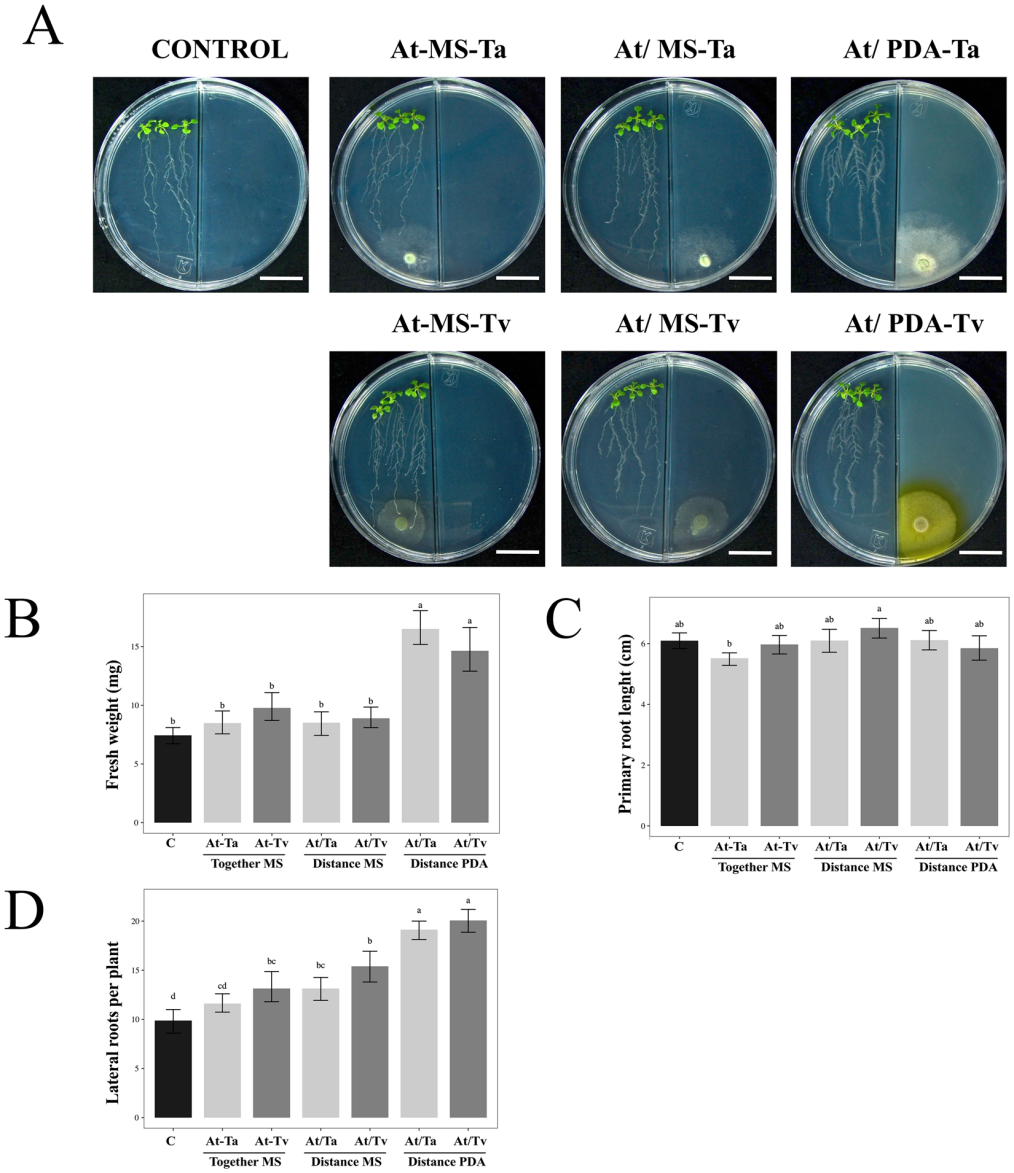
Effect of Arabidopsis growth with Trichoderma strains at 3 dpi. (**A**) Representative photographs of 13 day-old Arabidopsis (Col-0) plantlets grown on PDA or MS media after 3 dpi with *T. atroviride* (Ta) and *T. virens* (Tv). Trichoderma species were inoculated as follows: direct interaction (At-MS), split interaction without contact when the fungus was grown on MS medium (At/MS), split interaction without contact when the fungus was grown on PDA medium (At/PDA), and as control plantlets grown on MS without any inoculation. The scale bar corresponds to 2 cm. Physiological parameters were examined: (**B**) fresh weight (mg) (*n* = 15), (**C**) primary root length (*n* = 15), and (**D**) number of lateral roots per plant (*n* = 15). Barplots present mean ± SD values of dependent variables according to our experimental treatments. Letters refer to bootstrap pairwise two-sample robust tests, treatments without shared letters are significantly different.

**Figure 2:**
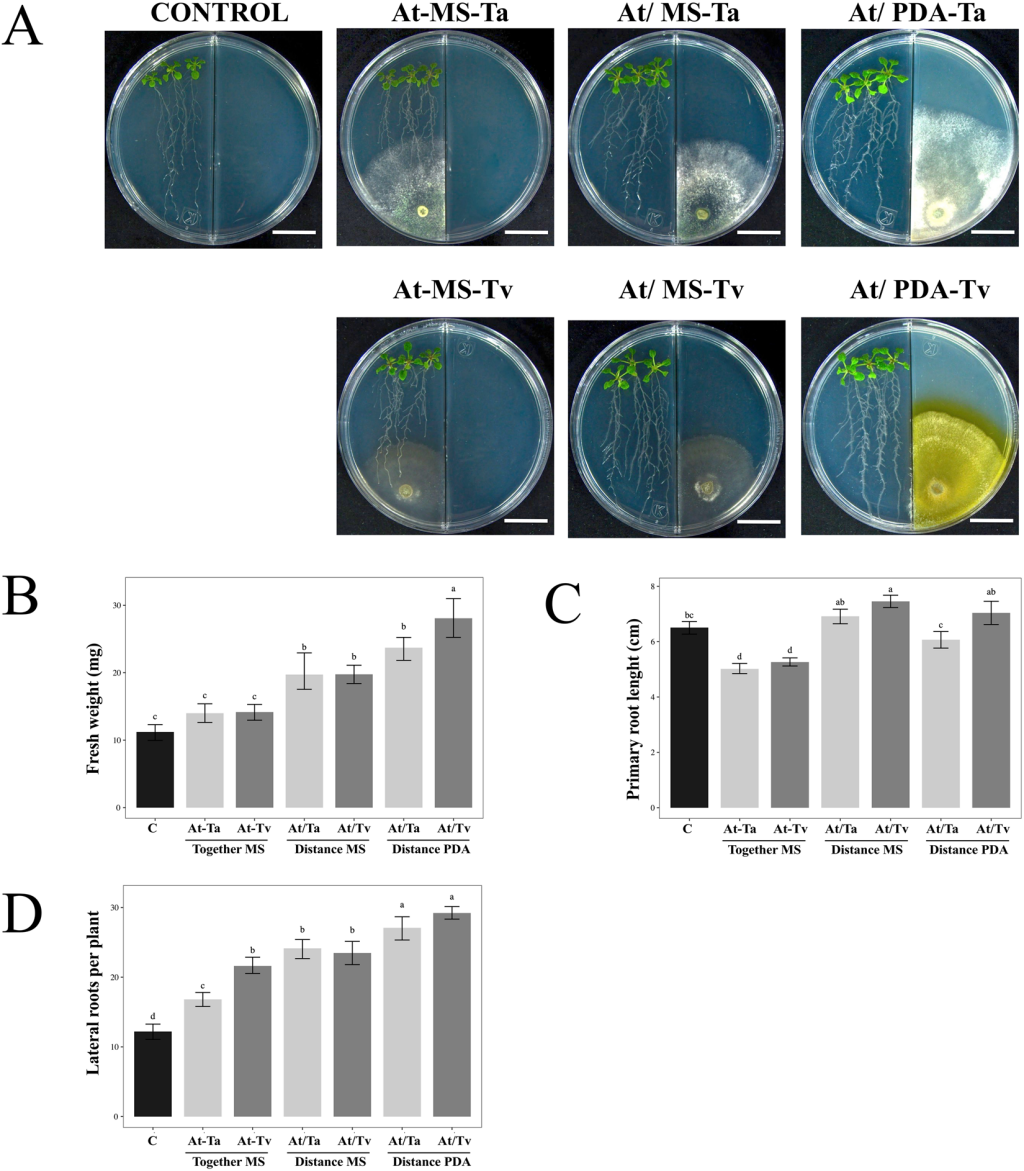
Effect of Arabidopsis growth with Trichoderma strains at 5 dpi. (**A**) Representative photographs of 13 day-old Arabidopsis (Col-0) plantlets grown on PDA or MS media after 5 dpi with *T. atroviride* (Ta) and *T. virens* (Tv). Trichoderma species were inoculated as follows: direct interaction (At-MS), split interaction without contact when the fungus was grown on MS medium (At/MS), split interaction without contact when the fungus was grown on PDA medium (At/PDA), and as control plantlets grown on MS without any inoculation. The scale bar corresponds to 2 cm. Physiological parameters were examined: (**B**) fresh weight (mg) (*n* = 15), (**C**) primary root length (*n* = 15), and (**D**) number of lateral roots per plant (*n* = 15). Barplots present mean ± SD values of dependent variables according to our experimental treatments. Letters refer to bootstrap pairwise two-sample robust tests; treatments without shared letters are significantly different.

In order to assess the plant promotion effect on Arabidopsis when Trichoderma is grown in different culture media, four experimental conditions were analyzed as follows: (i) direct or contact interaction (At-MS), (ii) split interaction without contact when fungus was grown on MS medium (At/MS), (iii) split interaction without contact when fungus was grown on PDA medium (At/PDA), and (iv) control plants grown on MS without inoculation (Figs 1A and 2A). Control plates of Arabidopsis seedlings growing on PDA were not included, because the plant did not develop well on this culture medium. Physiological parameters at 3 and 5 days post inoculation (dpi), such as fresh weight, main root length, and lateral roots number, were used to evaluate the effect of *T. atroviride* (Ta) and *T. virens* (Tv) on Arabidopsis growth (Figs 1 and 2).

At 3 dpi, we noticed an increment in the Arabidopsis fresh weight when Trichoderma was grown on PDA medium in the split system (*F* = *96.42*; *P* < 0.001; Suppl. Table 1), both *T*. *atroviride* and *T*. *virens* stimulated plant growth, which reached double the fresh weight in comparison to un-inoculated control plantlets (Fig. 1A,B). At this point in the interaction, we did not observe any differences in main root length (Fig. 1C) (*F* = 3; *P* < 0.0465; Suppl. Table 1). With respect to the lateral roots, the major difference was observed in the split system with both Trichoderma strains on PDA medium, in which the plants achieved nearly 20 lateral roots in contrast to the 10 lateral roots in the control in the absence of fungi (Fig. 1A,D) (*F* = 6.84; *P* < 0.001; Suppl. Table 1).

At 5 days of interaction, the increment of Arabidopsis fresh weight was higher with inoculated *T. virens* spores on PDA medium, obtaining 2.5 times more weight than the control (Fig. 2B) (*F* = 4.01; *P* < *0.001*; Suppl. Table 1). *Trichoderma atroviride* under the same conditions reached two times more than the un-inoculated plants. Also, the PDA growth condition of both fungal species generated the largest number of lateral roots in Arabidopsis seedlings, even higher than that obtained in the split interaction with MS medium (Fig. 2D) (*F* = 42.19; *P* < *0.001*; Suppl. Table 1). The main root length was promoted by *T. virens* under split system when the fungus was grown in MS medium (Fig. 2C) (*F* = 132.85; *P* < 0.0001; Suppl. Table 1), while the interactions in direct contact with fungi caused inhibition in main root growth (Fig. 2C).

### The* DR5:uidA* GUS signal increases under Trichoderma split interaction

**Figure 3:**
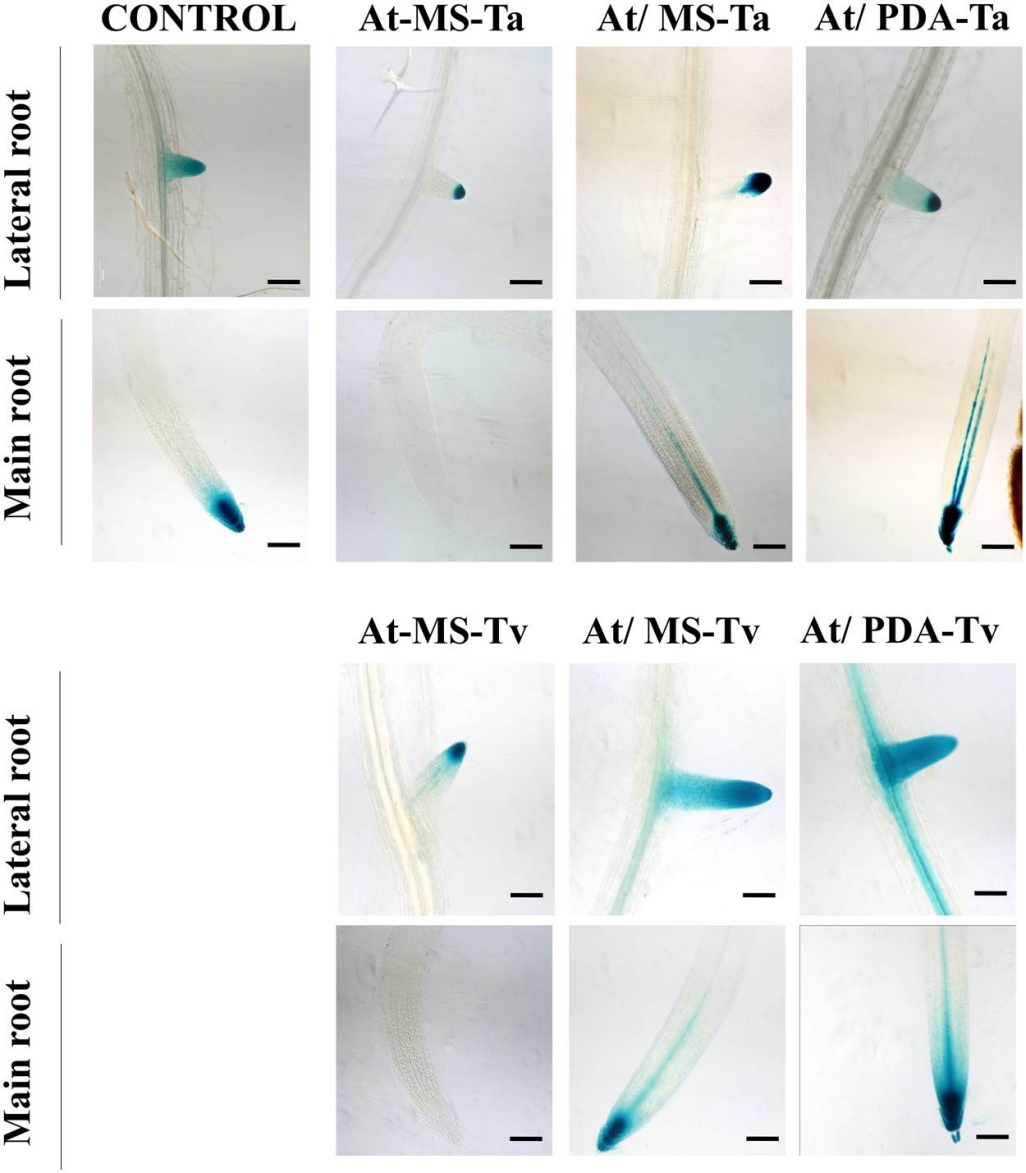
Effect of Trichoderma inoculation on expression of *DR5:uidA* at 22 °C. Seven day-old *DR5:uidA* Arabidopsis seedlings were inoculated with *T. atroviride* (Ta) *and T. virens* (Tv) spores on MS and PDA media for 5 days. The following conditions were analyzed: direct interaction (At-MS), split interaction without contact when the fungus was grown on MS medium (At/MS), split interaction without contact when the fungus was grown on PDA medium (At/PDA), and as control plantlets grown on MS without any inoculation. GUS histochemical analysis was performed for each treatment. Photographs are representative of individuals from at least 15 stained GUS seedlings. The scale bar corresponds to 100 µm. Images of main and lateral roots were acquired in a Zeiss Axio Imager M2 microscope with DIC contrast at 10x magnification, and processed in ZEN software.

The *DR5:uidA* line has been used to monitor sites of auxin accumulation^19^. Seven-day-old *DR5:uidA* seedlings were inoculated with *T. atroviride* (Ta) and *T. virens* (Tv) spores in the four different conditions mentioned above. After 5 dpi, the changes in GUS expression patterns were evaluated (Fig. 3). *T. atroviride* and *T. virens* in contact with the plant abated GUS expression in the primary root (Fig. 3), but expression was observed in the lateral roots tips. However, in the split interactions with both Trichoderma strains was detected an augment in GUS signal in the tip of primary root. In the case *T. virens* split interactions on both culture media an increase in GUS expression was not only observed in the primary root tip but also in the elongation zone and lateral roots (Fig. 3). These findings suggest that auxin accumulation is enhanced in the split interaction with *T. virens*.

### Trichoderma species growing in PDA are able to rescue the *rhd6* root-hair defective phenotype

**Figure 4:**
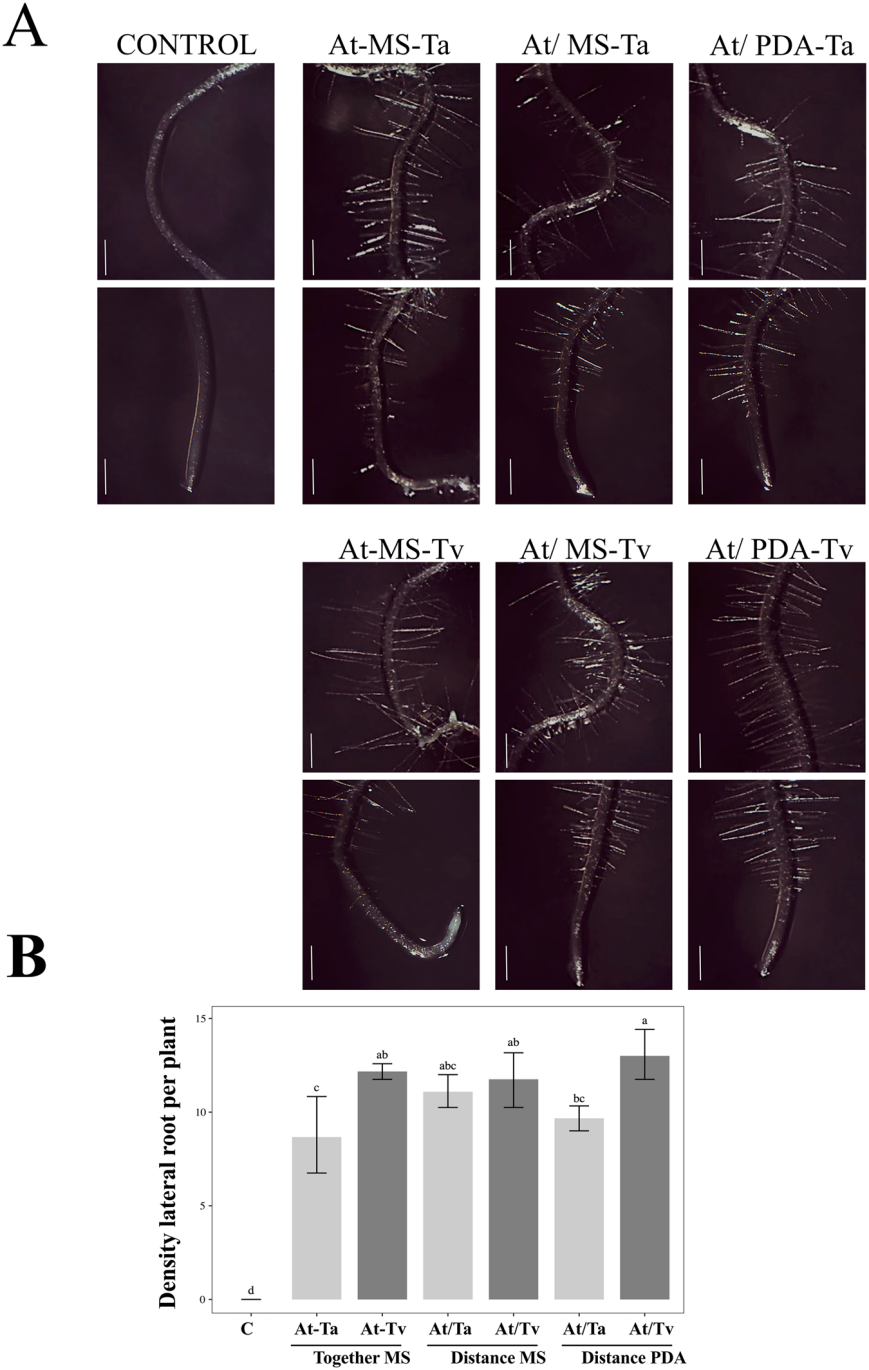
Effect of Trichoderma on root-hair formation in *rhd6* mutant. (**A**) 3 day-old *rhd6* mutant seedlings were inoculated with *T. atroviride* (Ta) and *T. virens* (Tv) for 5 days under the follow conditions: direct interaction (At-MS), split interaction without contact when the fungus was grown on MS medium (At/MS), split interaction without contact when the fungus was grown on PDA medium (At/PDA), and as control plantlets grown on MS without inoculation. Images were taken under a stereoscopic microscope (Motic smz-143 series) and are representative individuals from 10 seedlings. The scale bar corresponds to 500 µm. (**B**) Root hairs were counted in a region of 500 to 1000 μm from the main root tip. Density data were expressed in 500 μm area represented as mean ± SE (*n* = 10). Letters refer to bootstrap pairwise two-sample robust tests, treatments without shared letters are significantly different.

It has been previously reported that ethylene- and auxin-related mutations display alterations in root hair development^20^. This is the case for the *rhd6* mutant. This mutant is defective in root hair formation, and the mutant phenotype can be suppressed by the addition of auxin (indole-3-acetic acid) or the ethylene precursor 1-aminocyclopropane-1-carboxylic acid to the growth media^21^. We challenged both Trichoderma species to rescue the Arabidopsis *rhd6* root-hair defective phenotype in split and contact interactions. In Fig. 4A, it can be seen that in the three interaction conditions the root-hair defective phenotype of the *rhd6* mutant reverted in the presence of both fungal strains. Notably the highest density of root hairs was obtained when *T. virens* was grown on PDA in split interaction (Fig. 4B) (*F* = 1.93E + 29; *P* < *0.001*; Suppl. Table 2). These data reaffirm the fact that when Trichoderma grows in an optimal medium, it is able to enhance the benefits to plants.

### Volatile organic compounds (VOCs) production in Arabidopsis-Trichoderma interaction

In order to identify the VOCs secreted during the interaction Arabidopsis-Trichoderma, thirteen-day-old plantlets were exposed to *T. atroviride* and *T. virens* grown on MS medium (At/MS) or PDA medium (At/PDA) in split system during 5 days. As controls, we analyzed the VOCs secreted for 5 days under the following conditions: culture media (MS or PDA), Trichoderma strains grown on MS or PDA, and un-inoculated Arabidopsis plantlets grown on MS or PDA. The headspace VOCs were collected for 1 h and analyzed by GC-MS.

Different types of VOCs were found during the Arabidopsis-Trichoderma interaction, which were dependent of Trichoderma strain and culture medium (Tables 1 and 2). A total of 41 VOCs were detected comprising terpenes, alcohols, alkenes, ketones and aromatic compounds, being the terpenes the most abundant with 85.3%. Within the terpenes, the most abundant class was sesquiterpenes (SQTs). In the *T. virens*-Arabidopsis interaction, we observed that 100% of the VOCs were SQTs when the fungus was grown on MS; whereas when *T. virens* grown on PDA, SQTs represented 76.9% of the total VOCs (Table 1). On the other hand, we found that during the *T. atroviride*-Arabidopsis interaction, 50% of total VOCs identified correspond to SQTs in both media (Table 2).

**Table 1:** Volatile compound produced in T. *virens*-Arabidopsis interaction on MS and PDA medium under split system.

No.	Class	Compound	LRI^+^	Normalized amount of volatile compound (%)	Normalized amount of volatile compound (%)
				MS medium	PDA medium
1	Aromaticcompound	2-pentylfuran*	955		3.60 ± 2.11
2	Sesquiterpene (C15)	valencene	1349		10.32 ± 1.41
3	Monoterpene (C10)	cymene	1377		0.57 ± 0.29
4	Sesquiterpene (C15)	longifolene	1476	0.74 ± 0.37	
5	Sesquiterpene (C15)	α-bergamotene*	1486	7.40 ± 1.09	3.74 ± 2.15
6	Sesquiterpene (C15)	β-elemene	1488	7.48 ± 2.22	
7	Sesquiterpene (C15)	1β,4β,10β-guaia-5,11-diene	1561	1.73 ± 0.58	0.26 ± 0.17
8	Sesquiterpene (C15)	ledene	1601	14.46 ± 2.63	5.77 ± 1.38
9	Sesquiterpene (C15)	γ-cadinene	1606	36.99 ± 7.56	
10	Sesquiterpene (C15)	γ-muurolene	1608	1.17 ± 0.59	
11	Sesquiterpene (C15)	1,8-anhydro-*cis*-copaene	1625		2.37 ± 0.67
12	Sesquiterpene (C15)	germacrene D	1627	6.15 ± 1.45	
13	Sesquiterpene (C15)	β-selinene	1630	2.59 ± 2.01	
14	Sesquiterpene (C15)	α-selinene	1643		5.56 ± 1.01
15	Sesquiterpene (C15)	α-muurolene	1650	3.26 ± 0.62	
16	Sesquiterpene (C15)	α-chamigrene	1681		5.86 ± 1.02
17	Sesquiterpene (C15)	δ-cadinene	1684	17.38 ± 5.44	
18	Sesquiterpene (C15)	6α-cadina-4,9-diene	1686		37.26 ± 12.99
19	Sesquiterpene (C15)	*trans*-calamanene	1778	0.25 ± 0.13	
20	Sesquiterpene (C15)	α-calacorene	1867	0.41 ± 0.22	
21	Sesquiterpene (C15)	*epi*-bicyclosesquiphellandrene	1908		0.43 ± 0.25
22	Sesquiterpene (C15)	α-elemene*	2131		22.11 ± 9.33
23	Diterpene (C20)	isocembrene*	2230		2.15 ± 1.96

*Compounds produced by two Trichoderma strains growth in MS or PDA culture media during the interaction with Arabidopsis. The values are represented whit standard error (SEM), which were obtained by analyzing three biological replicas.

^**+**^Linear retention index.

**Table 2:** Volatile compound produced in T. *atroviride*-Arabidopsis interaction on MS and PDA medium under split system.

No.	Class	Compound	LRI^+^	Normalized amount of volatile compound (%)	Normalized amount of volatile compound (%)
				MS medium	PDA medium
1	Ketone	2-heptanone	842	1.94 ± 0.97	7.21 ± 0.30
2	Monoterpene (C10)	4(10)-thujene	875	29.75 ± 5.21	3.30 ± 2.21
3	Aromaticcompound	2-pentylfuran*	955	2.72 ± 1.61	2.27 ± 0.83
4	Ketone	2-nonanone	1222		0.69 ± 0.05
5	Alcohol	1-octen-3-ol	1332	1.77 ± 0.60	
6	Ketone	cyclopenten-2-one	1438	18.35 ± 11.47	
7	Sesquiterpene (C15)	α-bergamotene*	1486	4.29 ± 2.15	9.25 ± 2.92
8	Ketone	2-undecanone	1515		0.20 ± 0.04
9	Sesquiterpene (C15)	γ-curcumene	1553		0.46 ± 0.22
10	Unknown	unknownfrom lime oil	1561	2.81 ± 0.24	0.14 ± 0.06
11	Sesquiterpene (C15)	aromadendrene + epizonarene	1590	5.51 ± 0.36	0.24 ± 0.11
12	Sesquiterpene (C15)	italicene	1601	2.01 ± 0.22	0.78 ± 0.16
13	Sesquiterpene (C15)	γ-1-cadinene	1606		0.19 ± 0.07
14	Sesquiterpene (C15)	β-bisabolene	1664	0.23 ± 0.12	0.23 ± 0.01
15	Sesquiterpene (C15)	longipinene	1712	3.59 ± 0.02	2.24 ± 0.40
16	Aromaticcompound/alcohol	phenylethyl alcohol	1888		1.61 ± 1.49
17	Sesquiterpene (C15)	guaiene	2023	1.11 ± 0.59	
18	Sesquiterpene (C15)	cedr-8-ene	2106		0.31 ± 0.03
19	Sesquiterpene (C15)	α-gurjunene	2113	8.15 ± 0.91	1.37 ± 1.09
20	Sesquiterpene (C15)	α-elemene*	2131	3.24 ± 1.69	
21	Pyranone	6-pentyl-2H-pyran-2-one	2161	1.41 ± 0.50	30.06 ± 13.10
22	Diterpene (C20)	isocembrene*	2230	12.97 ± 5.19	38.66 ± 6.40

*Compounds produced by two Trichoderma strains growth in MS or PDA culture media during the interaction with Arabidopsis. The values are represented with standard error (SEM), which were obtained by analyzing three biological replicas.

^**+**^Linear retention index.

To determine whether plant and/or fungi are responsible for the production of the SQTs, we analyzed the production of VOCs by each organism. A total of 47 VOCs were identified in both Trichoderma strains grown on MS or PDA media. From the composition of the VOC mixture, the SQTs represented 77.7% in MS and 80% in PDA for *T. virens*, while for *T. atroviride* the SQTs correspond to 46.6% in MS and 42.1% in PDA (Suppl. Tables 3 and 4). Regarding Arabidopsis seedlings without interaction with fungi, the production of SQTs was not detected (Suppl. Table 5). These data showed that Trichoderma strains are responsible for SQTs production.

During the interaction of Arabidopsis with both Trichoderma strains only four compounds were in common, such as 2-pentylfuran, α-bergamotene, isocembrene, and α-elemene, although its production was dependent on the culture medium (Tables 1 and 2). In the Arabidopsis-*T. atroviride* interaction were detected twelve compounds in common with both culture media (Table 2). Particularly, we observed that *T. atroviride* grown on PDA showed a higher amount of isocembrene, and 6-pentyl-2H-pyran-2-one (6-PP), while on MS 4(10)-thujene and cyclopenten-2-one were the most abundant of the total mixture.

In the Arabidopsis - *T. virens* interaction (Table 1), VOCs production showed a differential profile dependent of the culture medium, for example with MS γ-cadinene and δ-cadinene compounds were more abundant; while, on PDA 6α-cadina-4,9-diene, α-elemene and valencene were the most representative volatile compounds. Only three compounds in common were found between the two culture medium in *T. virens* interaction, α-bergamotene, 1β,4β,10β-guaia-5,11-diene, and ledene (Table 1). Therefore, these results showed that culture medium and Trichoderma strains influenced the differential production of volatile compounds during the Arabidopsis-Trichoderma interaction.

### Growth of Arabidopsis with Trichoderma under low temperature

**Figure 5:**
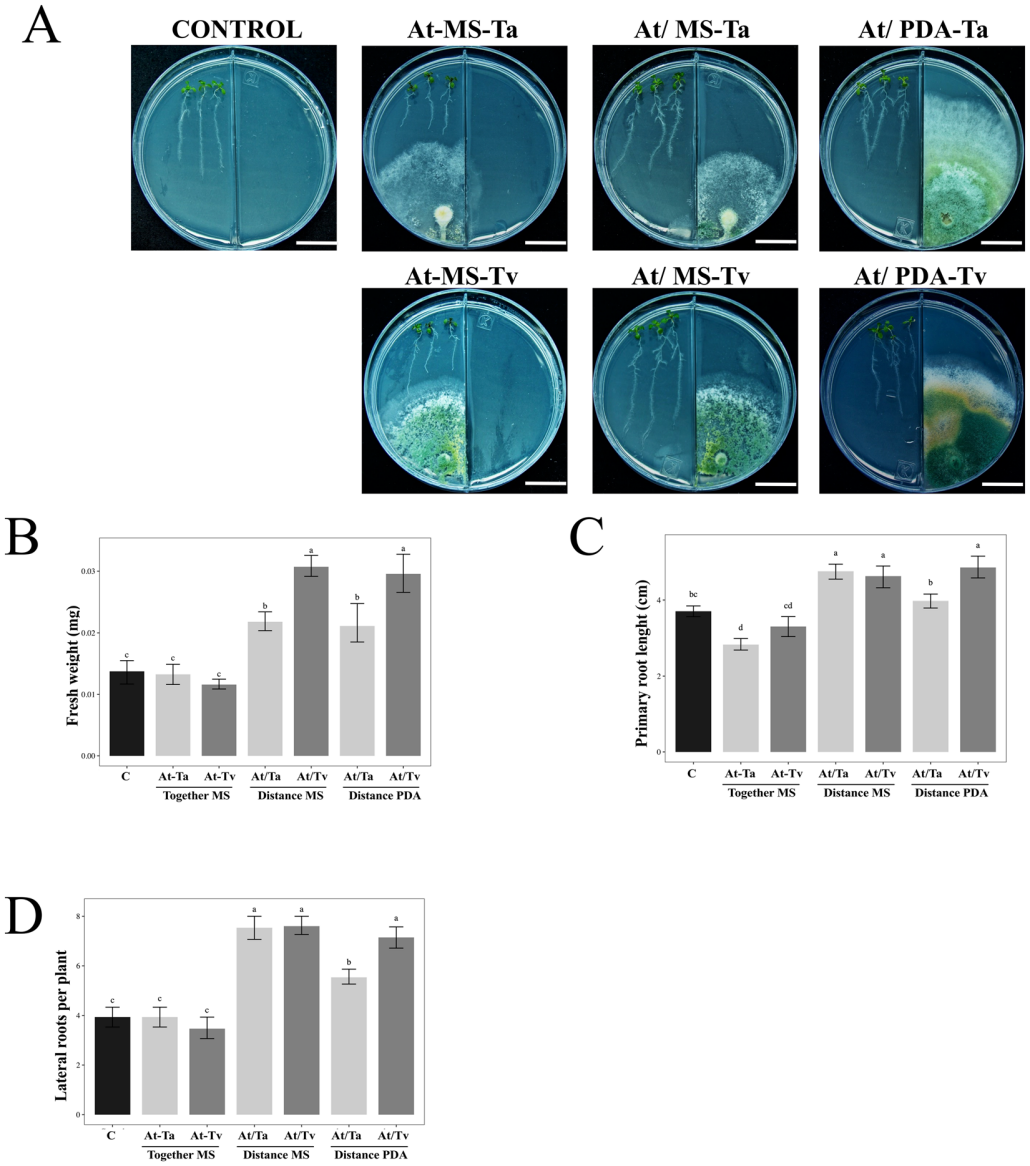
Trichoderma-Arabidopsis interactions at low temperature. (**A**) *T. atroviride* (Ta) and *T. virens* (Tv) strains were inoculated in the direct and split system at 12 °C (scale bar corresponds to 2 cm). The phenotype was evaluated at 12 dpi. Physiological parameters were measured (**B**) fresh weight (*n* = 5), (**C**) primary root length (*n *= 15), and D) number of lateral roots per plant (*n* = 15); un-inoculated plants were used as a control. Barplots present mean ± SD values of dependent variables according to our experimental treatments. Letters refer to bootstrap pairwise two-sample robust tests, treatments without shared letters are significantly different.

In order to evaluate whether Trichoderma strains were able to improve the vegetal growth under low temperatures (12 °C), seven-day-old Arabidopsis Col-0 seedlings were analyzed under the four different conditions (Fig. 5). Physiological parameters such as fresh weight, main root length, and lateral roots number were acquired at 12 dpi with Arabidopsis interacting with *T. atroviride* (Ta) and *T. virens* (Tv) (Fig. 5). Under cold treatment, we noticed an improved growth of Arabidopsis in the split interactions on MS and PDA medium (At-MS and At-PDA) in comparison to the control plantlets. Conversely, in the contact interaction experiment with both Trichoderma strains, slight plant damage in aerial part and slow growth were observed (Fig. 5A). Analysis of the fresh weight revealed an increase when *T. virens* interacted by split form, growing on MS or PDA media, obtaining around 90% more fresh weight than the control plantlets (Fig. 5B), and with *T. atroviride* we observed increase in 40% more fresh weight than the control and contact direct interactions (Fig. 5B) (*F* = 16.95; *P* < *0.001*; Suppl. Table 6). Primary roots were longer in the split interaction with *T. virens,* the length increased by 12% on MS and PDA media, but with *T. atroviride* an increase was only observed when the fungus was grown on MS medium (12%, Fig. 5C) (*F* = 10.23; *P* < *0.05*; Suppl. Table 6). Lateral roots were more abundant in the Trichoderma-Arabidopsis interaction over a distance with both strains under cold treatment (Fig. 1D). In this regard, the treatments that induced the greatest number of lateral roots were *T. virens* on MS and PDA and *T. atroviride* on MS, while *T. atroviride* on PDA had only a minor effect (*F* = *4.26; P* < *0.05*; Suppl. Table 6). It is important to mention that at 12 °C, *T. atroviride* grown on PDA was the treatment that resulted in less development in the plant.

**Figure 6:**
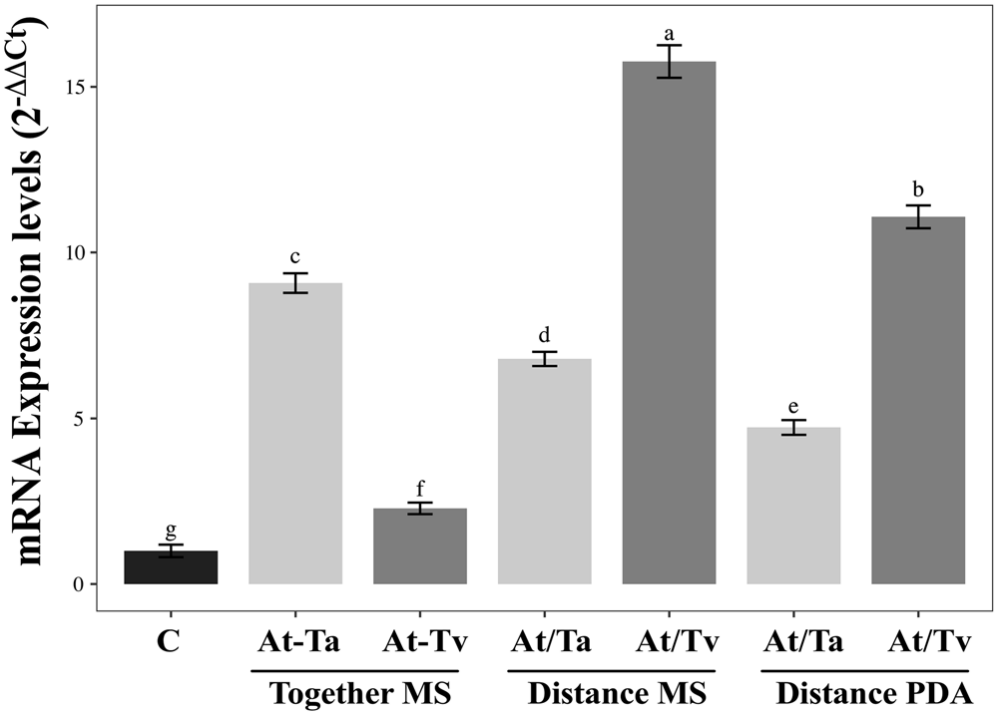
Analysis of *A. thaliana ERD14* expression levels in interaction with Trichoderma strains. qRT-PCR was performed on the different interactions at 6 h under 12 °C. Normalized fold change was calculated by comparing the *ERD14* gene expression level with a control (un-inoculated) after normalization to the Arabidopsis UBQ5 gene using the (2^−ΔΔCt^) method. Three biological replicates were analyzed with their respective technical replicates. Barplots present mean ± SD values of dependent variables according to our experimental treatments. Letters refer to bootstrap pairwise two-sample robust tests, treatments without shared letters are significantly different.

The expression pattern of *ERD14* gene, which is a cold-related dehydrin, was evaluated on the Arabidopsis-Trichoderma interactions at low temperature. An induction of the *ERD14* gene in all Trichoderma-Arabidopsis interactions was observed (Fig. 6). Interaction with *T. virens* at a distance induced significant increase in *ERD14* transcript in comparison to the direct interaction, reaching 15-fold on MS medium and 12.5-fold on PDA medium relative to the control (Fig. 6) (*F* = 54.3; *P* < *0.001*; Suppl. Table 7).

## Discussion

In nature, plants are not isolated organisms, so that throughout their evolution they have adapted to interact with diverse communities of symbiotic, beneficial, and pathogenic microorganisms^22^. It has been demonstrated some of these beneficial microorganisms carry out some crucial plant biological functions^23^. Some Trichoderma species are able to establish a plant beneficial interaction, which results in the promotion of plant growth and increased resistance against diseases^24^.

Recently, a few studies have reported that some species of Trichoderma are able to promote plant development without physical contact between fungus and plant^13,15,16^. Specifically, we reported that *T. virens* and *T. atroviride* have a higher impact on the development of Arabidopsis when the interaction was performed at distance, in a split system, in comparison with the direct contact interaction^9^. However, in the split system interaction Trichoderma was grown in MS media, which is used for vegetal *in vitro* growth^9^.

In the present study, we evaluated the effect of Trichoderma on *A. thaliana* development when the fungus was grown in an optimal fungal culture medium, such as potato dextrose agar (PDA). When both Trichoderma species were grown on PDA medium, major growth and development of the Arabidopsis plantlets were observed in comparison to the control condition and the others interactions with MS medium. Particularly, the number of lateral roots was higher in the PDA experiment condition with both Trichoderma species.

Microorganisms emit a broad spectrum of volatile organic compounds (VOCs) with diverse functions in plants such as defense, development, modulation of root architecture, and in microbe-plant communication^25,26^. The production of VOCs is influenced by different conditions such as nutrient content, microbial community composition, temperature, humidity, and pH^27,28^. It has been proposed that plant growth promotion in the split Trichoderma interaction can be attributed to the VOCs emitted by the fungus^13,15,16^.

Here, we identified a total of 41 VOCs in the Arabidopsis-Trichoderma split interaction, of which the majority were sesquiterpenes (SQTs). When we analyze the VOCs generated only by one of them, either by the plant or by the fungus, we noticed that the SQTs were originated by the Trichoderma strains. The production of SQTs by *T. virens* and *T. atroviride* has been recently reported^16^; authors analyzed the VOCs when both strains were grown on MS medium without plant interaction, noticing that majority of the VOCs produced by *T. virens* were SQTs 82.8%, in comparison with *T. atroviride* that only generate 31.2% of these compounds. Our data denote that the SQTs could be the main compounds that generate positive effects on Arabidopsis with *T. vires* and *T. atroviride* split interactions. In this sense, has been reported that during the interaction of Arabidopsis and Populus with *Laccaria bicolor*, the majority of the VOCs produced by this ectomycorrhizal fungus were SQTs, and the authors demonstrated that SQTs were the biologically active agents that triggered Arabidopsis and Populus lateral root formation^29^. Besides, the authors showed that *Cenococcum geophilum*, an ectomycorrhizal ascomycete that did not produce SQTs, did not promote lateral roots.

It is worth mentioning that only 4 of the 41 VOCs were in common between both Trichoderma strains, such is the case of 2-pentylfuran, α-bergamotene, isocembrene, and α-elemene. The production of 2-pentylfuran has been previously reported in *Bacillus megaterium*^30^, this compound had a positive effect on plant growth^30^. In our study, 2-pentylfuran was produced in both cultures with *T*. *atroviride*, and for *T*. *virens* in PDA. Previous reports have detected the production of α-bergamotene by Trichoderma and mycorrhizal fungi^15,16,31–34^, and we found that both Trichoderma strains produced α-bergamotene in MS and PDA cultures, which makes it a common compound for these fungi.

Among the compounds identified in the mixtures coming from the Arabidopsis-*T. atroviride* interaction is the 6-pentyl-2H-pyran-2-one (6-PP), which was more abundant in PDA (30.06%) than in MS (1.41%) medium. It has been reported that 6-PP promotes the development and regulates the morphogenesis of the roots in *A. thaliana*, although at high concentrations it can inhibit the growth of the main root^34^. This agrees with the observed at 5 dpi under *T*. *atroviride* split interaction on PDA media, we observed an inhibition in Arabidopsis primary root in comparison with the other split conditions, which could be due to a high accumulation of 6PP in Arabidopsis-*T*. *atroviride* PDA split interaction.

Data from this study support that VOCs production depends on the strain and the culture medium. In this sense, we observed that *T. virens* generated different VOCs when it was grown in MS or PDA, in comparison with *T. atroviride* that showed less variability in the composition of VOCs. It has been reported that the increment of certain secondary metabolites in Trichoderma species is influenced by the addition of particular substrates in the growth medium^35^. For example, *T. virens* increased IAA levels when the amino acid Tryptophan was added to the culture medium^10,35^. It is important to note that the generation of VOCs for each strain of Trichoderma and the culture medium could explain the changes observed in the growth and development of Arabidopsis.

When the Arabidopsis-Trichoderma interaction was exposed to a low temperature (12 °C), we observed a positive impact on the growth of Arabidopsis plantlets in the split system interaction in both types of culture media. Again, *T. virens* was the species that promoted major Arabidopsis growth when the fungal strain was inoculated in both types of media (PDA and MS) at 12 °C. Furthermore, we evaluated the expression of *ERD14* gene in Arabidopsis Col-0 subjected to cold stress with different Trichoderma interactions. *ERD14* is known to increase its expression level when plants are subjected to low temperatures^36^. We observed an induction of *ERD14* gene in all Trichoderma-Arabidopsis interactions analyzed, achieving the highest expression of the *ERD14* with *T. virens* in the split interaction. This suggests that some VOCs emitted by this Trichoderma species have the ability to trigger a higher expression of this gene in Arabidopsis plants subjected to cold stress, which was directly reflected in a higher tolerance to this type of stress.

In order to evaluate the Trichoderma effect on auxin accumulation in root tips, we analyzed the Arabidopsis *DR5:uidA* line under all interactions conditions. In the direct contact interaction, it was observed that both strains of Trichoderma abate the GUS signal in the main root tips, even in the assay at 12 °C. This could be attributed to the secondary metabolite called Trichokonin VI (TK VI), isolated from *T. longibrachiatum*, which is believed to inhibit main root growth^37^. The authors demonstrated that application of Trichokonin VI causes a decrease in auxin accumulation at the root tip, as well as a redistribution of auxins to the elongation zone. All of the above can be associated with an increase in the number of lateral roots that is a characteristic phenotype of the interactions between Trichoderma and plants. In this sense, we analyzed whether the inhibition of the GUS signal at the main root tips in the DR5 reporter line depended on the distance at which the fungus was inoculated in the direct contact interaction. For this, we performed an experiment in large Petri dishes (15 × 1.5 cm) in which the fungus was inoculated in the inferior part of the plate at 8 cm of the main root tip of Arabidopsis. It was observed that GUS reporter expression was not abated (Supplementary Fig. 1). From this result we can suggest that the diffusible compounds, responsible for the inhibition of root elongation, have not yet reached the root tip, or that their concentrations are low due to the distance between the fungus and Arabidopsis roots. In the split system when both fungi were grown on PDA and MS we observed a higher GUS expression in main and lateral root tips of *DR5:uidA* reporter line than the control plants; generally, the GUS signal was more abundant with *T. virens*. The results above reinforce the fact that the composition of the culture medium in which the fungus grows is a determining factor in metabolites production, and hence can be involved in auxin biosynthesis, redistribution, and transport.

In addition, we analyzed whether the *rhd*6 characteristic phenotype could be recovered during the interaction with *T. atroviride* and *T. virens*. In the direct and split interactions, both fungi were able to recover the deficiency in root hair formation in the *rhd6 *mutant. Remarkably, *T. virens* grown on PDA in the split interaction displayed the highest density of root hairs. It is known that the *rhd*6 characteristic phenotype can be recovered by the exogenous addition of auxins or ethylene^21^. Likewise, the *rhd*6 phenotype can be restored by the exogenous addition of auxin derivates such as IAAld and Iet isolated from *T. virens*^10^. In this sense is possible that some VOCs produced by Trichoderma could be auxin precursors or participate in auxin signaling pathway. Our results suggest that during the split interaction the VOCs produced by Trichoderma could induce auxins accumulation and redistribution in roots. For example, it has been reported that 6-pentyl pyrone (6-PP) could act as a growth regulator, particularly as an auxin inducer in lateral root primordia^34,38–40^. The production of the volatile compound 6-PP is related to the yellow pigmentation and coconut aroma characteristic of some Trichoderma species^40^. In addition, the involvement of 6-PP in plant defense activation on pea stems, tomato, and canola seedlings were reported^39^. Therefore, the 6-PP is a molecule that participates in root architecture and defense responses.

In this study, we learned that each species of Trichoderma produce different types of volatile organic compounds, and that the generation of these volatile compounds is related to the culture medium in which the fungus was grown, resulting in positive effects in plant growth.

## Materials and Methods

### Plant Material and Growth Conditions

In this study, we used the following *Arabidopsis thaliana* seeds*: DR5:uidA* reporter line^19^, the *rhd6* root-hair defective mutant^21^ and Arabidopsis WT ecotype Col-0. Seeds were vernalized for two days at 4 °C; subsequently, these were surface sterilized with 1 mL of chlorine solution at 40% (v/v) for 5 min and then rinsed six times in sterile distilled water. Agar plates containing 0.2x Murashige and Skoog (MS) medium^41^ [0.2x basal medium w/vitamins, 0.75% (w/v) sucrose, 1% (w/v) agar, pH 7.0] were used to germinate and grow the seeds. Afterwards, the plates were incubated in a growth chamber with a photoperiod of 16 h of light/8 h darkness, light intensity of 120 µmol m^−2^ s^−1^ and temperature of 22 ± 1 °C for the time required in each experiment described below.

### Fungal growth and inoculum preparation

*Trichoderma atroviride* (IMI 206040; Ta) and *T. virens* (Gv29.8; Tv) were the fungal strains used to carry out the interaction with Arabidopsis plantlets. Each fungus was grown on Potato Dextrose Agar (PDA, Difco) plates for eight days at 28 °C. Then, the spore suspension was obtained in sterile distilled water. Total conidia were quantified in a Neubauer chamber under 40x magnification in a Motic model BA-300 microscope. For each treatment, we inoculated 1 µL per plate of a suspension of 1 × 10^6^ spores per µL.

### Trichoderma-Arabidopsis interaction on MS and PDA at 22 °C and 12 °C

For the interaction assays, we used five plates with three plantlets, which were transferred to the left side of divided Petri dishes (9.0 × 1.5 cm). Four interaction conditions were evaluated: i) plantlets without inoculum were used as a control, ii) plantlets were inoculated directly at the bottom of the left side of the plates (At-MS-Ta and At-MS-Tv), iii) plantlets were inoculated with the fungus at a distance on MS medium (i.e. on the right side of the split plate, At/MS-Ta and At/MS-Tv), and iv) plantlets were inoculated with the fungus at a distance on PDA medium (i.e. on the right side of the divided plate, At/PDA-Ta and At/PDA-Tv). The interaction experiment at 22 °C was carried out with Arabidopsis Col-0 plantlets of 13 day-old, and analyzed at 3 and 5 dpi. For the assay at 12 °C, we used 7 day-old Col-0 plantlets for Trichoderma spore inoculation, and data were collected at 12 dpi. Photographs shown are representative of the experiment conducted with five biological replicates. These experiments were repeated at least 3 times with similar results.

### Estimation of Arabidopsis growth parameters under Trichoderma interaction

Arabidopsis plantlets subjected to interaction with Trichoderma at different temperatures were analyzed. Principal root length of 15 plantlets per treatment (*n* = *15*) was evaluated using the IMAGE J software (http://rsb.info.nih.gov/). The estimation of the number of emerged lateral roots (stage of development as reported by Malamy and Benfey^42^) was obtained by counting each lateral root in all of the plantlets in the interaction with each fungus. For the interaction at 22 °C the fresh weight (mg) per plant was obtained on an analytical balance (*n* = *15*). In the case of the interaction at 12 °C, the fresh weight values were obtained as follows: the fifteen plantlets of each treatment were divided into groups of three plantlets (*n* = *5*), due to the small size of the plantlets, and subsequently weighed on an analytical balance.

### Histochemical analysis of *DR5:uidA* Arabidopsis line

We inoculated 1 µL per plate (1 × 10^6^ spores) in 7-day-old *DR5:uidA* seedlings for the four interaction conditions previously described. After 5 dpi, the *DR5:uidA* plantlets were subjected to GUS histochemical analysis as previously reported^43^. After 16 h of incubation at 37 °C in a GUS reaction buffer (0.5 mg/ml of 5-bromo-4-chloro-3indolyl-β-D-glucuronide in 100 mM sodium phosphate, pH 7) plantlets were clarified as described by Malamy and Benfey^42^. For each interaction condition, at least 15 plantlets (3 plantlets per plate) were analyzed. Clarified plantlets were placed on a glass slide and coated with coverslips. Images of primary and lateral roots were acquired with a Zeiss Axio Imager M2 microscope with DIC contrast at 10X magnification and processed in ZEN software. Photographs shown are representative of the experiment conducted with five biological replicates. This experiment was repeated at least 3 times with similar results.

### Root hair density determination of the *rhd6* mutant

Arabidopsis seedlings of *rhd6* mutant^21^ were subject to direct and split interactions with *T. atroviride* and *T. virens*. Spores of Trichoderma (1 µL at 1 × 10^6^) were inoculated at 3-day-old in the four different interaction systems previously described. The primary root zone was photographed at 5 dpi using a stereoscopic microscope (Motic smz-143 series). Root hairs were counted in a region of 500 to 1000 μm approximately from the primary root tip. Ten plants were measured for each treatment (*n* = 10). This experiment was repeated at least 3 times with similar results.

### RNA isolation and quantitative real-time PCR (qRT-PCR)

For the qRT-PCR analysis, Arabidopsis Col-0 ten-day-old plantlets were subjected to the four interaction conditions with Trichoderma. One microliter of Trichoderma spores (1 × 10^6^) were inoculated at 22 °C for 3 days in order to allow the fungus to grow before the low-temperature assay. The plates were left at 12 °C for 6 h, and subsequently plantlets were frozen in liquid nitrogen. Total RNA was isolated using the Concert Plant RNA reagent system following the manufacturer’s recommendations (Invitrogen, Carlsbad, CA, USA). DNase Turbo (Ambion, Austin, TX, USA) treatment was used to remove possible remaining genomic DNA. Quantitative RT-PCR (qRT-PCR) analysis was performed with 50 ng of total RNA by the one-step assay using the Power SYBR Green RT-PCR Mix kit (Applied Biosystems, USA). qRT-PCR was performed using the Step One Real-Time PCR Detection System (Applied Biosystems) in a 10 μL reaction mixture containing 5 μL of Power SYBR Green RT-PCR Mix (2X), 200 nM of each primer, and 0.08 μl of RT Enzyme Mix (125X). The thermal cycling conditions used were reported previously^44^. For each RNA sample, three biological replicates (*n = *3) were analyzed with their respective technical replicates. Quantitation was based on a cycle threshold value^45^; the mRNA transcript level of the *ERD14* (At1g76180) gene was compared to the *A. thaliana UBQ5* gene (At3g62250). The primers used were ERD14-Fw GTAGAGGAGGAGAAGAAAGAT, ERD14-Rv GCCAACAAACTTACACATCAC and UBQ5-F 5′ GGAGTGCCCTAACGCAACC 3′ and UBQ5-R 5′ GCTACAACAGATCAAGCTTCAAC 3′ for the *UBQ5* gene loading. For primer amplification efficiency, we carried out a standard curve with serial dilutions of RNA^46,47^, using the qRT-PCR thermal conditions described above. For UBQ5 primer pair the slope was-3.25, resulting in 102.95% amplification efficiency, and for ERD14 the slope was-3.307 resulting in 100.9% amplification efficiency.

### Volatile organic compounds (VOCs) analysis by headspace GC-MS

Presence of different VOCs of *T. atroviride* and *T. virens* in interaction with Arabidopsis were analyzed using a gas chromatograph GC-7890b (Agilent technologies, Santa Clara, California, USA) coupled to a mass spectrophotometer EM-5977A (Agilent technologies, Santa Clara, California, USA) fitted with a HP-Innowax Polyethylene glycol phase capillary GC Column (30.0 m × 0.320 mm i.d. × 0.25 μm, Agilent technologies, Santa Clara, California, USA) and a manual-sampler for solid phase micro-extraction SPME (Supelco Analytical Bellafonte PA USA). A small hole was made in each Petri dish (1.5 mm diameter) with a sterile drill, to allow the SPME fiber entrance. Two experimental conditions were analyzed as follows: i) split interaction without contact when fungus was grown on MS medium (At/MS-Ta), ii) split interaction without contact when fungus was grown on PDA medium (At/PDA-Tv). As controls, three conditions were analyzed: i) VOCs produced by Trichoderma strains growth on MS and PDA without interaction with Arabidopsis plantlets, ii) VOCs produced by un-inoculated Arabidopsis plantlets grown in MS (At/MS) or PDA (At/PDA) media, and iii) the background of VOCs secreted by culture media (MS or PDA). We analyzed three plates for each condition (*n = 3*). Three plantlets of 13 day-old per plate were placed on the side where the hole was made, afterward were inoculated with 1 µL (1 × 10^6^) spores of Trichoderma. After, plates were sealed perfectly with parafilm paper to avoid the escape of the VOCs and were incubated at 22 °C for five days. VOC-analysis was performed according to Stoppacher^32^ with some modifications. An SPME fiber assembly polydimethylsiloxane/divinylbenzene 65 mm was utilized to extract the VOCs; the SPME fiber was introduced in the small hole of the Petri dishes and exposed for 1 h. After injection, the compounds were desorbed for 20 min in a splitless injector at 200 °C. The oven temperature was held at 40 °C for 10 min, after temperature increased 3 °C per minute to reach 180 °C and then held at this temperature for 10 min. Helium was used as carrier gas at a constant flux of 1.5 mL/min. Compounds were identified by a deconvolution using the W10N11 database (Wiley10Nist11) and based on the linear retention index (LRI) values^48^, which were calculated after analyzing C_6_ and C_25_ *n*-alkanes.

### Statistical analysis

Data from factorial experiments were evaluated with mixed effects ANOVAS including interaction terms. Fixed effects included Medium (MS vs PDA), interaction (Trichoderma position relative to Arabidopsis: adjacent, distance and control) and Trichoderma (*T. atroviride* vs *T. virens*). Petri dish was included as random term to avoid auto-correlation and pseudo-replication. Presented results are minimum adequate models, which were obtained following a backward stepwise selection procedure started from full maximum models, including all second degree interaction terms. Non-significant terms were discarded based upon likelihood ratio tests and Akaike Information Criterion^49,50^. Normality of dependent variables as well as heteroscedasticity and normality of model residuals were checked visually by normal q-q plots, histograms and by using the Shapiro–Wilk test where necessary^51^. Post-hoc Letters in barplots that separate significantly different groups were drawn from Pairwise Two-Sample Robust Tests with confidence intervals based upon 599 bootstrap samples (function pairwise Robustb Test of the rcompanion library; Mangiafico)^52^. False discovery rates of multiple pairwise testing were controlled with the Benjamini, Hochberg and Yekutieli p adjustment method^53^. Petri dish was used as a grouping factor to avoid pseudo-replication. All analyses were realized using R version 3.4.1^54^.

## Electronic supplementary material

Supplementary Information
