# Supplementary material for: The Arabidopsis-Trichoderma interaction reveals that the fungal growth medium is an important factor in plant growth induction
Source: Sci Rep. 2018 Nov 6;8:16427. doi: 10.1038/s41598-018-34500-w (PMC6219587; doi:10.1038/s41598-018-34500-w)
Supplement: Supplementary file 1 — Supplementary Information [file 41598_2018_34500_MOESM1_ESM.doc]

**The Arabidopsis-Trichoderma interaction reveals that the fungal growth medium is an important factor in plant growth induction**

González-Pérez Enrique^1^, Ortega-Amaro María Azucena^1^, Salazar-Badillo Fátima Berenice^1^, Elihú Bautista^2^, David Douterlungne^3^ and Jiménez-Bremont Juan Francisco^1*^

**Supplementary Table 1| Mixed effects ANOVA results of fresh weight, primary root length and lateral roots in Arabidopsis-Trichoderma interaction at 3 and 5 dpi 22 °C. Fixed effects included Medium (MS vs PDA), interaction (Arabidopsis)**

|  |  |  |  |
| --- | --- | --- | --- |
|  | ***Df*** | ***F*** | ***Pr(>F)*** |
| **Fresh weight at 3 dpi** |  |  |  |
| Medium | 1 | 96.42 | 0.001 |
| Residuals | 33 |  |  |
| **Primary root length at 3 dpi** |  |  |  |
| Interaction | 2 | 2.96 | 0.0675 |
| Medium: Trichoderma | 3 | 3 | 0.0465 |
| Residuals | 29 |  |  |
| **Lateral roots per plant at 3 dpi** |  |  |  |
| Interaction | 2 | 49.58 | 0.001 |
| Medium | 1 | 52.08 | 0.001 |
| Trichoderma | 1 | 6.84 | 0.001 |
| Residuals | 30 |  |  |
| **Fresh weight at 5 dpi** |  |  |  |
| Medium | 1 | 97.28 | 0.001 |
| Trichoderma | 2 | 9.78 | 0.0006 |
| Interaction | 1 | 21.30 | 0.0001 |
| Medium:Trichoderma | 1 | 4.01 | 0.0546 |
| Residuals | 29 |  |  |
| **Primary root length at 5 dpi** |  |  |  |
| Medium | 1 | 4.76 | 0.0375 |
| Trichoderma | 2 | 9.71 | 0.0006 |
| Interaction | 1 | 132.85 | 0.001 |
| Medium: Trichoderma | 1 | 3.56 | 0.0694 |
| Residuals | 29 |  |  |
| **Lateral roots per plant at 5 dpi** |  |  |  |
| Medium | 1 | 114.80 | 0.001 |
| Trichoderma | 2 | 42.19 | 0.001 |
| Interaction | 1 | 23.57 | 0.001 |
| Residuals | 30 |  |  |

**Supplementary Table 2| Values of *F* and *P* of density root hairs per plant in Arabidopsis *rhd6*** mutant during Arabidopsis-Trichoderma interaction

|  |  |  |  |
| --- | --- | --- | --- |
|  | ***Df*** | ***F*** | ***Pr(>F)*** |
| **Lateral root per plant** |  |  |  |
| Interaction | 1 | 1.33E+29 | *0.001* |
| Medium | 1 | 7.55E+26 | *0.001* |
| Trichoderma | 1 | 1.02E+30 | *0.001* |
| Interaction: Trichoderma | 1 | 8.15E+28 | *0.001* |
| Medium: Trichoderma | 1 | 1.93E+29 | *0.001* |
| Residuals | 66 |  |  |

**Supplementary Table 3| Volatile compound produced by *Trichoderma virens* on MS and PDA medium**

|  | | | |  |  |
| --- | --- | --- | --- | --- | --- |
| **No.** | **Type of compound** | **Compound** | **LRI** | **Normalized amount of volatile compound (%)** | **Normalized amount of volatile compound (%)** |
|  |  |  |  | **MS medium** | **PDA medium** |
| 1 | Alcohol/ketone | 3-hydroxy-2-butenone | 1050 |  | 1.62 ± 0.81 |
| 2 | Alkene | 1,3-octadiene | 1308 |  | 3.34 ± 0.87 |
| 3 | Alkene | 2,3-dimethyl-1,3-pentadiene | 1366 |  | 0.16 ± 0.09 |
| 4 | Sesquiterpene (C15) | Caryophyllene | 1469 |  | 1.21 ± 0.36 |
| 5 | Sesquiterpene (C15) | β-elemene | 1488 |  | 3.06 ± 0.60 |
| 6 | Sesquiterpene (C15) | δ-gurjunene | 1561 |  | 0.61 ± 0.45 |
| 7 | Sesquiterpene (C15) | α-gurjunene + β-panasinsene | 1595 |  | 16.14 ± 2.44 |
| 8 | Sesquiterpene (C15) | cycloisosativene | 1600 |  | 1.08 ± 0.28 |
| 9 | Sesquiterpene (C15) | ledene | 1601 | 11.56 ± 9.68 | 0.26 ± 0.13 |
| 10 | Sesquiterpene (C15) | δ-cadinene | 1606 |  | 11.08 ± 7.68 |
| 11 | Sesquiterpene (C15) | δ-muurolene | 1608 | 2.60 ± 1.80 | 3.82 ± 1.72 |
| 12 | Sesquiterpene (C15) | germacrene D | 1627 | 9.43 ± 8.84 |  |
| 13 | Sesquiterpene (C15) | β-selinene | 1630 |  | 1.22 ± 0.15 |
| 14 | Sesquiterpene (C15) | α-selinene | 1643 | 11.05 ± 9.62 | 1.51 ± 0.19 |
| 15 | Sesquiterpene (C15) | α-muurolene | 1650 |  | 4.00 ± 1.99 |
| 16 | Sesquiterpene (C15) | γ-gurjunene | 1657 |  | 1.35 ± 0.45 |
| 17 | Sesquiterpene (C15) | γ-cadinene | 1684 | 11.74 ± 4.18 |  |
| 18 | Sesquiterpene (C15) | 6α-cadina-4,9-diene | 1686 | 27.59 ± 14.14 | 17.45 ± 3.50 |
| 19 | Sesquiterpene (C15) | α-calacorene | 1867 | 2.89 ± 0.98 |  |
| 20 | Aromatic compound/alcohol | phenetyl alcohol | 1888 | 16.26 ± 8.17 | 11.39 ± 3.04 |
| 21 | Sesquiterpene (C15) | Copaene | 1909 |  | 1.29 ± 0.68 |
| 22 | Sesquiterpene (C15) | 2-isopropyl-5-methyl-9-methylene[4.4.0]dec-1-ene | 2011 |  | 18.84 ± 2.00 |
| 23 | Pyranone | 6-pentyl-2H-pyran-2-one | 2161 | 6.89 ± 6.63 |  |
| 24 | Sesquiterpene (C15) | germacrene B | 2218 |  | 0.56 ± 0.29 |

***LRI (**Linear Retention Index)

**Supplementary Table 4| Volatile compounds produced by *Trichoderma atroviride* on MS and PDA medium**

|  |  | | | | |
| --- | --- | --- | --- | --- | --- |
| **No.** | **Class** | **Compound** | **LRI** | **Normalized amount of volatile compound (%)** | **Normalized amount of volatile compound (%)** |
|  |  |  |  | **MS medium** | **PDA medium** |
| 1 | Alcohol | 2-butanol | 718 |  | 1.86 ± 0.95 |
| 2 | Ketone | 2-heptanone | 842 |  | 0.97 ± 0.88 |
| 3 | Alcohol | isomylalcohol | 915 |  | 24.61 ± 3.62 |
| 4 | Aromatic compound | 2-pentyl-furan | 955 | 3.12 ± 1.62 | 2.86 ± 1.31 |
| 5 | Alcohol/ketone | 3-hydroxy-2-butanone | 1050 |  | 2.39 ± 0.17 |
| 6 | Alcohol | 1-octen-3-ol | 1332 | 4.88 ± 2.46 |  |
| 7 | Alcohol | 2-ethyl-1-hexanol | 1355 | 6.42 ± 0.54 |  |
| 8 | Sesquiterpene (C15) | α-bergamotene | 1486 | 2.69 ± 1.38 | 10.10 ± 0.59 |
| 9 | Ketone | 2-undecanone | 1515 | 1.45 ± 0.77 | 0.63 ± 0.17 |
| 10 | Ketone | 3-ethyl-2-cyclopenten-1-one | 1534 |  | 0.36 ± 0.04 |
| 11 | Sesquiterpene (C15) | γ -curcumene | 1553 | 5.89 ± 4.52 |  |
| 12 | Monoterpene (C10) | γ-terpinene | 1555 |  | 0.13 ± 0.02 |
| 13 | Unknown | Unknown from lime oil | 1561 | 8.05 ± 4.02 | 0.22 ± 0.06 |
| 14 | Sesquiterpenes (C15) | aromadendrene + epizonarene | 1590 | 4.56 ± 1.65 |  |
| 15 | Sesquiterpenes (C15) | italicene | 1601 | 0.94 ± 0.47 | 1.00 ± 0.16 |
| 16 | Sesquiterpenes (C15) | γ-1-cadinene | 1606 |  | 0.44 ± 0.06 |
| 17 | Sesquiterpenes (C15) | β-bisabolene | 1664 |  | 0.25 ± 0.13 |
| 18 | Sesquiterpenes (C15) | guaiene | 2023 | 4.50 ± 0.85 |  |
| 19 | Sesquiterpenes (C15) | cedr-8-ene | 2106 |  | 1.73 ± 1.00 |
| 20 | Sesquiterpenes (C15) | α-gurjunene | 2113 | 8.79 ± 0.74 | 0.65 ± 0.33 |
| 21 | Sesquiterpenes (C15) | α-elemene | 2131 | 7.33 ± 0.42 | 0.43 ± 0.06 |
| 22 | Pyranone | 6-pentyl-2H-pyran-2-one | 2161 | 29.33 ± 1.23 | 33.86 ± 2.80 |
| 23 | Sesquiterpene (C15) | β-cedrene | 2190 |  | 1.77 ± 0.64 |
| 24 | Diterpene (C20) | cembrene | 2230 | 6.21 ± 1.35 | 6.93 ± 0.65 |
| 25 | Aromatic compound | 2-ethylhexylsalicilate | 2297 | 5.84 ± 4.10 |  |

***LRI (**Linear Retention Index)

**Supplementary Table 5| Volatile compounds produced by *A. thaliana* on MS and MS-At/PDA medium**

|  |  |  |  |  |  |
| --- | --- | --- | --- | --- | --- |
| **No.** | **Type of compound** | **Compound** | **LRI** | **Normalized amount of volatile compound (%)** | **Normalized amount of volatile compound (%)** |
|  |  |  |  | **MS medium** | **MS-At/PDA medium** |
| 1 | Aromatic compound | xylene | 764 | 1.72 ± 0.58 | 2.36 ± 0.04 |
| 2 | Aromatic compound | styrene | 991 | 62.88 ± 2.61 | 33.75 ± 8.98 |
| 3 | Aldehyde | nonanal | 1230 | 29.45 ± 0.64 | 50.08 ± 11.62 |
| 4 | Aromatic compound/aldehyde | furfural | 1345 | 4.56 ± 1.13 | 13.81 ± 2.68 |
| 5 | Aromatic compound | 3,5-dioctoxyphenol | 2005 | 1.40 ± 0.25 |  |

***LRI (**Linear Retention Index)

**Supplementary Table 6| Values of *F* and *P* of fresh weight, primary root length and lateral roots per plant in Arabidopsis-Trichoderma interaction under cold stress at 12 °C**

|  |  |  |  |
| --- | --- | --- | --- |
|  | ***Df*** | ***F*** | ***Pr(>F)*** |
| **Fresh weight** |  |  |  |
| Medium | 1 | 29.18 | *0.001* |
| Trichoderma | 1 | 24.89 | *0.001* |
| Interaction | 1 | 116.22 | *0.001* |
| Medium: Trichoderma | 1 | 4.72 | *0.05* |
| Trichoderma: Interaction | 1 | 16.95 | *0.001* |
| Residuals | 24 |  |  |
| **Primary root lenght** |  |  |  |
| Medium | 1 | 43.28 | *0.001* |
| Trichoderma | 1 | 28.06 | *0.001* |
| Interaction | 1 | 294.81 | *0.001* |
| Medium:Trichoderma | 1 | 18.43 | *0.001* |
| Trichoderma:Interaction | 1 | 10.23 | *0.05* |
| Residuals | 84 |  |  |
| **Lateral root per plant** |  |  |  |
| Medium | 1 | 35.79 | *0.001* |
| Trichoderma | 1 | 13.45 | *0.001* |
| Interaction | 1 | 895.73 | *0.001* |
| Medium: Trichoderma | 1 | 63.87 | *0.001* |
| Trichoderma: Interaction | 1 | 4.26 | *0.05* |
| Residuals | 83 |  |  |

**Supplementary Table 7| Values of *F* and *P* of *ERD14* gene expression levels under cold stress during Arabidopsis-Trichoderma interaction**

|  |  |  |  |
| --- | --- | --- | --- |
|  | ***Df*** | ***F*** | ***Pr(>F)*** |
| **Lateral root per plant** |  |  |  |
| Interaction | 1 | 637.38 | *0.001* |
| Medium | 1 | 356.06 | *0.001* |
| Trichoderma | 1 | 379.99 | *0.001* |
| Interaction: Trichoderma | 1 | 2178.95 | *0.001* |
| Medium: Trichoderma | 1 | 54.3 | *0.001* |
| Residuals | 12 |  |  |

**Supplementary Figure 1.**

**Legend of Figure**

**Supplementary Figure 1.**Effect of Trichoderma inoculation on expression of *DR5:uidA* in a 150 mm Petri dishes. **A)** Representative photographs of seven d-old *DR5:uidA* Arabidopsis plantlets inoculated with *T. atroviride* and *T. virens*spores at the bottom of the plate and subjected to direct contact interaction for five days (At-MS) on 150 mm Petri dishes. The scale bar corresponds to 2 cm. **B**) Exemplary images of GUS staining on lateral and main roots from *DR5:uidA* Arabidopsis plantlets, uninoculated plants were used as a control. The scale bar corresponds to 100 µm. At least 15 plants were analyzed. Notably, the GUS signal expression was not abated during a long distance interaction between the Arabidopsis DR5:*uidA* reporter line and Trichoderma strains compared to the interaction conditions on 90 mm plates. Images of primary and lateral roots were acquired in a Zeiss Axio Imager M2 microscope whit DIC contrast and processed in ZEN software.
